# Upregulation of EMR1 (ADGRE1) by Tumor-Associated Macrophages Promotes Colon Cancer Progression by Activating the JAK2/STAT1,3 Signaling Pathway in Tumor Cells

**DOI:** 10.3390/ijms25084388

**Published:** 2024-04-16

**Authors:** Rokeya Akter, Rackhyun Park, Soo Kyung Lee, Eun ju Han, Kyu-Sang Park, Junsoo Park, Mee-Yon Cho

**Affiliations:** 1 Department of Global Medical Science, Yonsei University, Wonju College of Medicine, Wonju 26426, Republic of Korea; akterbinteha30@yonsei.ac.kr; 2 Department of Life Science, Yong-In University, Yongin 17092, Republic of Korea; flowblue@yongin.ac.kr; 3 Department of Physiology, Yonsei University, Wonju College of Medicine, Wonju 26426, Republic of Korea; katoolee@yonsei.ac.kr (S.K.L.); qsang@yonsei.ac.kr (K.-S.P.); 4 Department of Pathology, Yonsei University, Wonju College of Medicine, Wonju 26426, Republic of Korea; dmswntttt@naver.com; 5 Division of Biological Science and Technology, Yonsei University, Wonju 26426, Republic of Korea; junsoo@yonsei.ac.kr

**Keywords:** colon cancer, EMR1/ADGRE1, macrophages, JAK2/STAT1,3 signaling, lymph node metastasis

## Abstract

Previously, we reported that epidermal growth factor-like module-containing mucin-like hormone receptor-like 1 (EMR1/ADGRE1) is abnormally expressed in colon cancer (CC) and is a risk factor for lymph node metastasis (LNM) and poor recurrence-free survival in patients with abundant tumor-associated macrophages (TAMs). However, the signaling pathways associated with EMR1 expression in CC progression remain unclear. In this study, we aimed to explore the role of EMR1 and its signaling interactions with macrophages in CC progression. Spatial transcriptomics of pT3 microsatellite unstable CC tissues revealed heightened Janus kinase (JAK)/signal transducer and activator of transcription (STAT) signaling in EMR1-HL CC with LNM compared to EMR1-N CC without LNM. Through in vitro coculture of CC cells with macrophages, EMR1 expression by CC cells was found to be induced by TAMs, ultimately interacting with upregulated JAK/STAT signaling, increasing cell proliferation, migration, and motility, and reducing apoptosis. JAK2/STAT3 inhibition decreased the levels of EMR1, JAK2, STAT1, and STAT3, significantly impeded the proliferation, migration, and mobility of cells, and increased the apoptosis of EMR1^+^ CC cells compared to their EMR1^KO^ counterparts. Overall, TAMs-induced EMR1 upregulation in CC cells may promote LNM and CC progression via JAK2/STAT1,3 signaling upregulation. This study provides further insights into the molecular mechanisms involving macrophages and intracellular EMR1 expression in CC progression, suggesting its clinical significance and offering potential interventions to enhance patient outcomes.

## 1. Introduction

Colon cancer (CC) is a multifaceted and heterogeneous disease characterized by diverse genetic alterations and aberrations in signaling pathways that occur within tumor cells and in the intricate tumor microenvironment (TME). The interplay between these modifications within the signaling milieu of cancer cells and TME plays a pivotal role in driving cancer progression and metastasis [1,2,3]. The TME is a specialized biological microenvironment that encompasses various cells, including leukocytes, myeloid lineage cells, fibroblasts, endothelial cells, and their associated secreted components [4]. This complex microenvironment exerts multifaceted effects, fostering tumor growth, attenuating immune responses, promoting therapeutic resistance, and providing niches conducive to metastasis [5]. Consequently, deciphering the mechanisms through which the TME contributes to tumor progression could enable the development of efficacious drugs and preventive strategies for advanced CC.

In our previous study, the upregulation of epidermal growth factor-like module-containing mucin-like hormone receptor-like 1 (EMR1/ADGRE1) in cancer cells was found to be induced by tumor-associated macrophages (TAMs). Notably, this upregulation has a relationship with lymph node metastasis (LNM) and worse recurrence-free survival in patients with colorectal cancer (CRC) [6]. Therefore, EMR1 expression is a potential prognostic marker for TAM-rich CRC. EMR1 is a member of the adhesion G protein-coupled receptors (aGPCRs) and belongs to the epidermal growth factor-seven-transmembrane group-II-E (ADGRE) subfamily [7,8,9]. Initial investigations into the physiological mechanisms of aGPCRs were due to their impact on the immune system [10]; however, according to several recent studies, the dysregulation of normal expression may contribute to the development of various diseases, including different types of cancers [7,8]. Despite their significance, many aGPCRs remain orphan receptors and their signaling pathways have not been fully elucidated.

Over the past decade, extensive studies have been conducted to elucidate the activation signal transduction mechanisms of aGPCRs. Accordingly, some members of the aGPCRs family have gradually been recognized as modulators of proliferation, metastasis, and cancer cell communication in the TME of numerous cancers [11]. The overexpression of CD97/ADGRE5 in CRC, was found to enhance tumor metastasis by activating the β-catenin signaling pathway and its coupling to Gα12/13 [12,13,14]. GPR56/ADGRG1 promotes the conversion of tumor cells into mesenchymal cells, contributing to tumor growth and metastasis via the PI3K/AKT pathway, and predicting a worse prognosis for patients with CRC [15]. Furthermore, elevated ELTD1/ADGRL4 expression in patients with CRC correlates with LNM and unfavorable prognoses. However, the underlying signaling mechanism has not been elucidated [16], similar to that of the EMR1/ADGRE1 investigation performed in our previous study. EMR1 is normally expressed in myeloid cells, serves as a marker for macrophages and eosinophils, and regulates immune cell function [17,18]. Although abnormal EMR1 expression has been observed in CC, the intricate regulatory molecular mechanisms, downstream signal transduction pathways, and associated genes governing the expression and function of EMR1 in tumors, which may facilitate CC progression, remain elusive. By exploring the molecular mechanisms and signaling pathways involving intracellular EMR1 expression and macrophages in TME, we anticipate gaining an enhanced understanding of the clinical significance of intracellular EMR1 expression in CC progression.

In the current study, we conducted spatial transcriptomic profiling to determine the signaling pathway associated with EMR1 expression and LNM in surgically resected microsatellite unstable (MSI-H) CC. In addition, a coculture system involving EMR1^+^ or EMR1/ADGRE1 knockout (EMR1^KO^) CC cells with macrophages was employed to elucidate changes in genes expression and biological functions.

## 2. Results

### 2.1. Whole Transcriptomic Profiling by GeoMx

#### 2.1.1. Data Reduction for Comparative Spatial Transcriptomic Profiling of CC

In this study, 14 patients diagnosed with pT3 MSI-H CC who underwent curative-intent colon surgery were enrolled. All participants were >50 years old, and most tumors were predominantly in the right colon, with only one exception. Pearson’s chi-squared correlation analysis revealed a significant positive correlation between EMR1 expression in tumor cells and the presence of LNM. Other parameters, such as age, sex, and location, were not significantly correlated with LNM in CC. The clinicopathological details of the cohort are summarized in Table 1.

**Figure 1:**
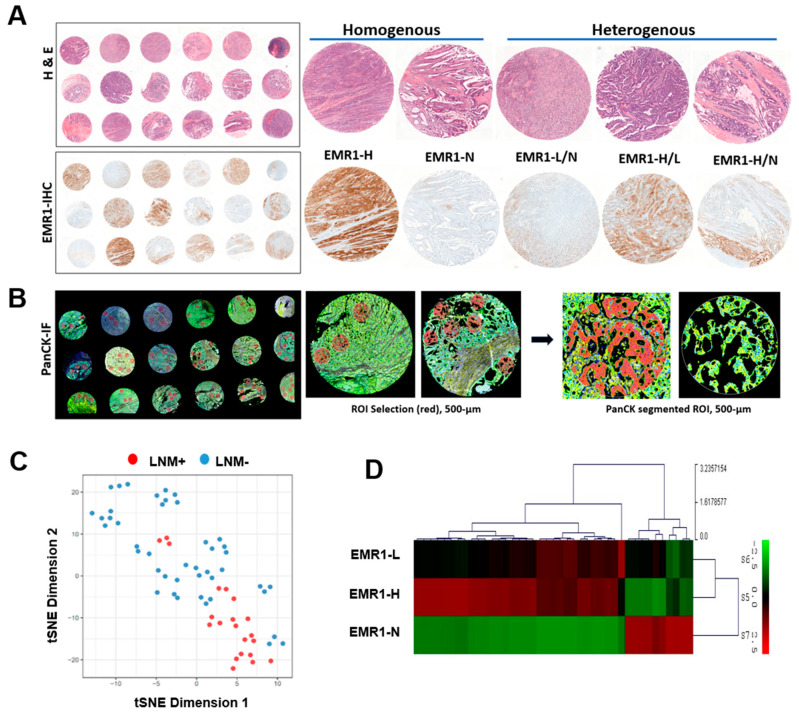
**ROI selection and data reduction in the spatial profiling of CC.** (**A**) Hematoxylin and eosin staining and EMR1-IHC staining of the TMA. Representative EMR1 expression pattern (homogenous vs. heterogenous) in IHC staining. (**B**) ROI selected from PanCK-IF staining based on EMR1-IHC expression for spatial profiling using the NanoString GeoMx digital spatial profiler assay. The morphology markers, PanCK (green, epithelial-cell marker) and Syto13 (blue, nuclear DNA), are indicated. Artificial overlay of tissue segmentation is indicated for each ROI to visualize the PanCK+ (red) segment in an area of 500 µm width (circled). (**C**) Dimensionality reduction visualization of all ROIs according to overall gene expression profiles based on tSNE. The tSNE plot was annotated by the PanCK segment based on the individual ROI from each CC case, according to LNM (LNM+ vs. LNM−). (**D**) Molecular profiling of major DEGs in EMR1-H, EMR1-L, and EMR1-N is illustrated as a heat map. Abbreviations: CC: colon cancer, ROI: region of interest, DEG: differentially expressed gene, PanCK: pan Cytokeratin, IHC: immunohistochemistry, IF: immunofluorescence, LNM: lymph node metastasis, H = high, L = low, N = negative, and TMA, tissue microarray.

To assess intratumoral transcriptional alterations in the epithelial compartment of the ROI, we separately investigated the cytokeratin-positive (PanCK+) fractions within each ROI (Figure 1A,B). Data exploration using dimension reduction by t-SNE revealed clear clustering of ROIs in individual cases according to LNM (LNM+ vs. LNM−) (Figure 1C). In the differential gene expression (DEG) analysis, EMR1-H exhibited an expression pattern similar to that of EMR1-L but different from that of EMR1-N (Figure 1D).

#### 2.1.2. Comparison of Differential Gene Expression in CC According to LNM Using Spatial Transcriptomic Profiling

**Figure 2:**
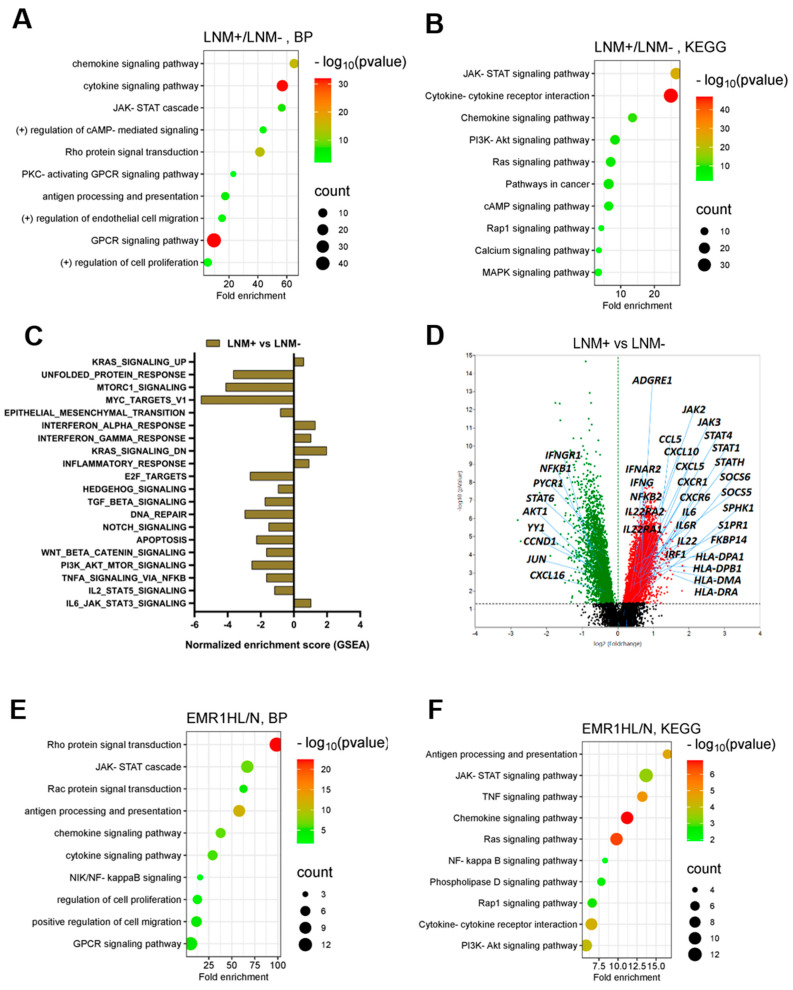

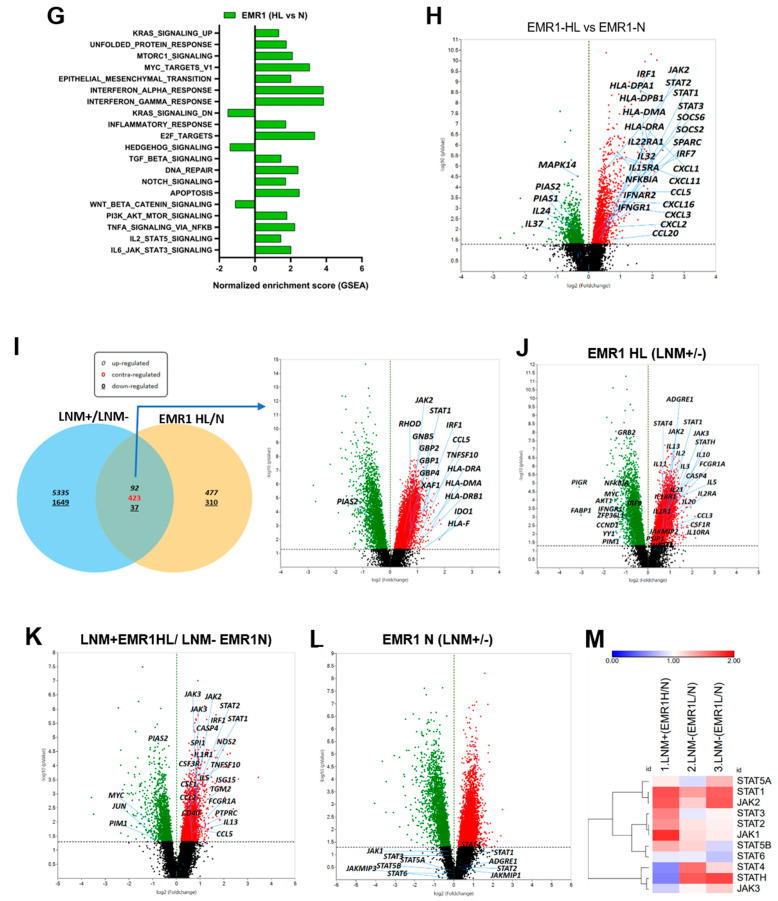
**Spatial transcriptomic analysis of CC according to LNM and EMR1 expression.** (**A,B**) GO-BP and KEGG pathway enrichment analyses of the DEGs in CC for the LNM+ vs. LNM- groups. The top 10 significantly enriched pathways are presented. The size of the dots represents the gene count, and the color of the dots represents the -log10 (*p*-value), the transition from red to green indicates a lower to higher *p*-value. (**C**) NES obtained from GSEA for the LNM+ vs. LNM- groups. (**D**) Volcano plot of DEGs, including significantly upregulated (red dots) and downregulated (green dots) genes between LNM+ and LNM- ROIs. (**E,F**) GO (GO Term BP) and KEGG pathway enrichment analyses of the DEGs in the EMR1-HL/N comparison group. A total of 10 significantly enriched pathways are presented. The size of the dots represents the gene count, and the color of the dots represents the -log10 (*p*-value); the transition from red to green color indicates a smaller to larger *p*-value. (**G**) NES obtained from GSEA for the EMR1-HL vs. EMR1-N groups. (**H**) Volcano plot of DEGs, including most significant fold-change DEGs between the EMR1-HL and EMR1-N comparison groups. (**I**) Venn diagram illustrating common transcriptome and exclusively expressed genes in the LNM+/LNM- and EMR1-HL/EMR1-N CC groups. (**J**–**L**) Volcano plots of some significant DEGs related to the JAK/STAT signaling pathway in the comparison groups with different combinations (EMR1+LNM). (**M**) Heat map analysis of JAK and STAT gene expression in EMR1 heterogeneous CC case; EMR1-H or EMR1-L area was compared with EMR1-N area in the same case. Abbreviations: CC; colon cancer, LNM; lymph node metastasis X = LNM+, Y = LNM−, HL = high low, N = negative, ROI: region of interest, DEG: differentially expressed gene, GO: gene ontology, KEGG: Kyoto Encyclopedia of Genes and Genomes database, BP: biological process, NES: normalized enrichment score, GSEA: gene set enrichment analysis.

In the DEG analysis, among the 18,675 genes, 3802 were significantly upregulated and 919 genes were significantly downregulated in the epithelial segment of LNM+ CC (fold > 1.5, normalized value (log2) = 4, *p* < 0.05) compared with that of CC without LNM. Based on GO biological process (GO-BP) and KEGG pathway enrichment analyses, 10 significant pathways, including the upregulation of JAK-STAT signaling, antigen presentation, and chemokine–cytokine-mediated signaling, were enriched in the LNM+ group compared to those in the LNM-CC group (Figure 2A,B, Supplementary Figure S1C). GSEA revealed significant enrichment of gene sets, including IL-6 JAK–STAT3 signaling, inflammatory response, IFN-α and IFN-γ response, and KRAS signaling pathways, in the LNM+ group compared to those in the LNM- group (Figure 2C, Supplementary Figure S1A). Notable genes, including ADGRE1, JAK2, JAK3, STAT1, STAT4, and STATH, were upregulated in the LNM+ group compared to the LNM- CC group (Figure 2D). Our comprehensive analysis provided integrated insights into the molecular landscape of LNM+ CC and highlighted key genes and pathways associated with LNM.

#### 2.1.3. Comparison of DEGs in CC According to EMR1 Expression Using Spatial Transcriptomic Profiling

In the comparative analysis of DEGs between the EMR1-HL and EMR1-N groups, 1651 significantly upregulated and 893 significantly downregulated genes were identified in the EMR1-HL group (fold >1.3, normalized value (log2) = 4, *p* < 0.05). Based on the DEGs in DAVID GO-BP and KEGG pathway enrichment analyses, 10 significantly enriched pathways were found in the EMR1-HL group. Notable pathways included positive regulation of JAK-STAT signaling; IFN-γ, NF-κB, and cytokine–chemokine signaling; and antigen presentation (Figure 2E,F, Supplementary Figure S1D). GSEA revealed significant enrichment of gene sets related to inflammatory response, IFN-α/γ response, IL-2 STAT5 signaling, IL-6-JAK–STAT3 signaling, KRAS signaling, TNF-α signaling via NF-κB, and apoptotic pathways in the EMR1-HL group compared to the EMR1-N group (Figure 2G, Supplementary Figure S1B). Several key genes, including *JAK2*, *JAK3*, *STAT1*, *STAT2*, and *STAT3,* were upregulated in the EMR1-HL group compared to those in the EMR1-N group (Figure 2H). Overall, our findings highlight the distinct expression patterns and enriched pathways associated with EMR1 expression, providing a comprehensive understanding of the molecular alterations linked to EMR1-HL compared to EMR1-N in CC.

#### 2.1.4. DEGs Involved in LNM and EMR1 Expression in CC

Among the overlapping DEGs, 92 were significantly upregulated, 37 were significantly downregulated, and 423 displayed contra-regulation in the EMR1-HL/N and LNM+/LNM- groups (fold > 1.3, normalized value (log2) = 4, *p* < 0.05) (Figure 2I). JAK-STAT signaling-related genes, including *JAK2*, *JAK3*, *STAT1*, *STAT2*, and *STAT4*, were significantly upregulated in the EMR1-HL group in LNM+ CC compared to EMR1-N and LNM- CC (Figure 2J,K,L). In CC cases with heterogeneous EMR1 expression, specific JAK-STAT genes (*JAK2*, *JAK3*, *STAT1*, *STAT2*, and *STAT3*) were upregulated in the EMR1-H/L ROI compared to those in the EMR1-N ROI within the same tumor (Figure 2M). The upregulation of JAK-STAT signaling-related genes suggests a potential role of this pathway in EMR1 expression and lymph node involvement in CC.

### 2.2. In Vitro Analysis of CC Cells Based on EMR1 Expression Using a Coculture System with Macrophages

#### 2.2.1. Molecular Changes between EMR1^+^ or EMR1^KO^ CC Cells Cocultured with Macrophages In Vitro Based on the nCounter System

**Figure 3:**
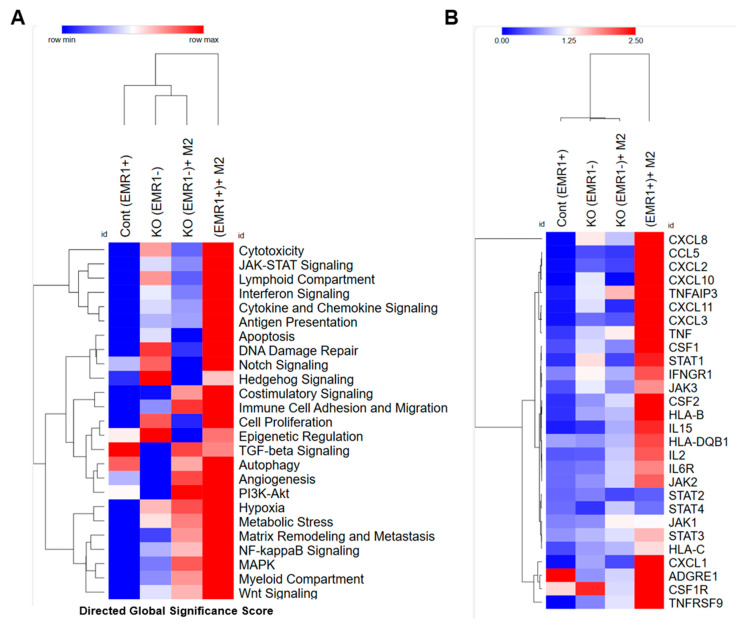
**Molecular changes between EMR1^+^ and EMR1^KO^ CC cells after coculture with macrophages in vitro.** (**A**) Heatmap of the Directed Global Significance Scores indicating the extent to which a given gene set was upregulated or downregulated relative to a given covariate. The selected covariates are listed along the right side of the heatmap. (**B**) Heatmap illustrating major DEGs between EMR1^+^ vs. EMR1^KO^ CC cells with or without macrophage coculture. Abbreviations: CC: colon cancer, DEG: differential expressed gene, KEGG: Kyoto Encyclopedia of Genes and Genomes database, KO: knockout.

Using the CRISPR-Cas9 lentiviral expression system, we successfully established an EMR1^KO^ in HCT15 CC cells, achieving a composition of 91.7% EMR1^KO^ cells following culture of a single isolated colony (Supplementary Figure S3A). THP1-derived M2 macrophages were polarized to >64.1% after stimulation with IL-4 and IL-13 (Supplementary Figure S3B). The direct global significance score obtained from the nCounter analysis revealed a distinct upregulation of key signaling pathways, including JAK/STAT signaling, apoptosis, interferon signaling, cytokine and chemokine signaling, and antigen presentation, in the EMR1^+^ group compared to those in the EMR1^KO^ group after coculture with macrophages (Figure 3A). DEG analysis using nCounter revealed a significant increase in the expression of JAK2, JAK3, STAT1, and STAT3 genes in EMR1^+^ CC cells after coculture with macrophages compared to those in EMR1^KO^ cells (Figure 3B). KEGG pathway analysis further revealed that the JAK/STAT signaling pathway is a crucial pathway associated with EMR1 expression in CC (Supplementary Figure S2A). Overall, our results highlight the changes in gene expression induced by EMR1 in CC cells, emphasizing the pivotal role of JAK/STAT signaling in the interplay between cancer cells and macrophages. These findings are consistent with the spatial profiling results and provide valuable insights into the molecular mechanisms underlying the EMR1-mediated effects in the TME.

#### 2.2.2. Biological Behavior and Molecular Changes of CC Cells Due to EMR1 Expression in a Coculture System with Macrophages

**Figure 4:**
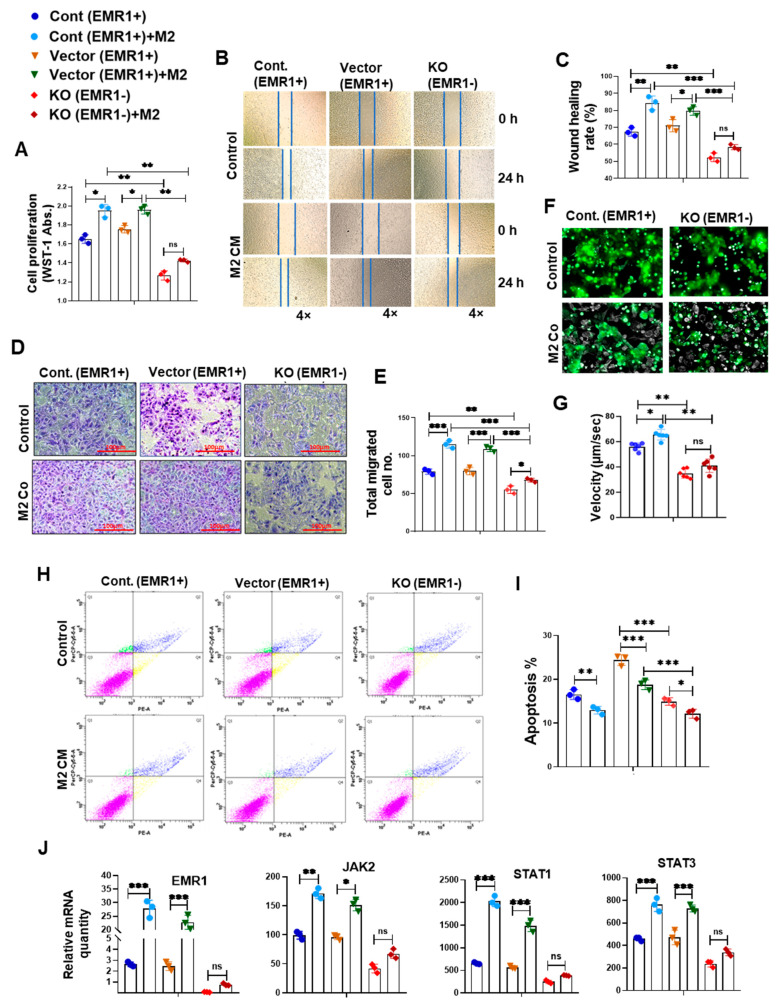

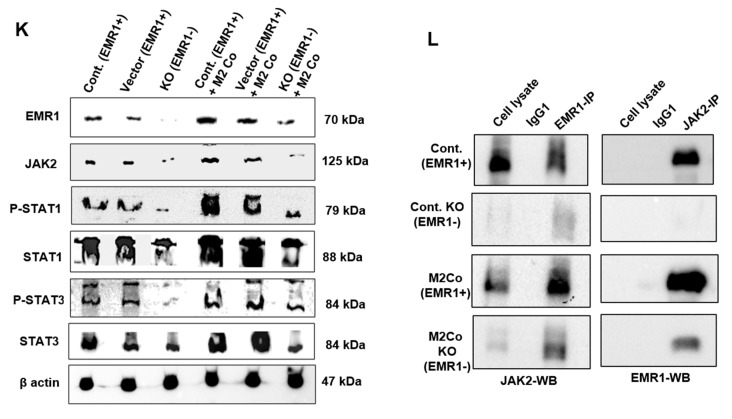
**EMR1 regulated the proliferation, migration, and motility of CC cells after coculture with macrophages in vitro.** (**A**–**I**) Results of the WST-1 (**A**), scratch wound healing (**B**,**C**), Transwell migration (**D**,**E**), live cell imaging using NanoLive (**F**,**G**) and flow cytometric analysis using the PE Annexin V based apoptosis kit (**H**,**I**) for the proliferation, migration, mobility, and apoptosis of EMR1^+^ and EMR1^KO^ CC cells cocultured with or without macrophages. (**J**) qRT-PCR was performed to determine the relative mRNA expression between EMR1^+^ and EMR1^KO^ CC cells cocultured with or without macrophages. (**K**) Western blot analysis was performed to determine the relative protein expression between EMR1^+^ and EMR1^KO^ CC cells cocultured with or without macrophages. (**L**) Co-immunoprecipitation (Co-IP) analysis was performed to determine the EMR1-JAK2 protein–protein interaction in EMR1^+^ and EMR1^KO^ CC cells cocultured with or without macrophages. The results represent two independent experiments, each comprising three replicates, with statistical analysis conducted based on these three replicates. Error bars represent SD. Statistical significance is indicated by * *p* < 0.033; ** *p* < 0.002; and *** *p* < 0.001 compared to the control using multiple Bonferroni one-way ANOVA. Abbreviations: CC-cont: colon cancer cell control, CM: conditioned media, Co: coculture, SD: standard deviation, ns: not significant. PE-A: PE-Annexin-V; and PerCP-Cy5-5-A: 7-aminoactinomycin D (7-AAD).

The WST-1 assay revealed significantly elevated proliferation of EMR1^+^ cells compared to EMR1^KO^ cells at baseline. Following treatment with macrophage-CM, EMR1^+^ cell proliferation significantly increased compared to the insignificant change in EMR1^KO^ cell proliferation (Figure 4A). Wound healing assays revealed enhanced migration of EMR1^+^ cells compared to EMR1^KO^ cells at 24 h post-scratching. After CM treatment, EMR1^+^ cell migration was significantly increased, whereas EMR1^KO^ cell migration remained unchanged (Figure 4B,C). Transwell migration assays further confirmed the increased migration of EMR1^+^ cells compared to EMR1^KO^ cells at 48 h post-seeding, with macrophages in the lower portion. Notably, EMR1^KO^ cell migration was not significantly altered (Figure 4D,E). Live cell imaging mobility analysis revealed a significantly higher migration velocity of EMR1^+^ cells than EMR1^KO^ cells. During the coculture with macrophages, EMR1^+^ cell velocity significantly increased, whereas EMR1^KO^ cell velocity remained unchanged (Figure 4F,G). Flow cytometric analysis using the PE Annexin V apoptosis kit revealed a significantly higher apoptotic rate (%) for EMR1^+^ cells than EMR1^KO^ cells. However, when treated with macrophage CM, the apoptotic rate (%) of both EMR1^+^ and EMR1^KO^ cells decreased significantly compared to culture without macrophages (Figure 2H,I). The *EMR1, JAK2, STAT1,* and *STAT3* genes and the p-STAT1 and p-STAT3 proteins were significantly upregulated after coculture with macrophages or CM treatment in EMR1^+^ cells compared to those in EMR1^KO^ cells. (Figure 4J,K). The qRT-PCR and Western blot analysis revealed that the *EMR1, JAK2, STAT1,* and *STAT3* mRNA level and proteins together with the p-STAT1 and p-STAT3 proteins were significantly upregulated after coculture with macrophages in EMR1^+^ cells compared to those in EMR1^KO^ cells. (Figure 4J,K). The Co-IP experiments revealed binding between EMR1 and JAK2 in EMR1^+^ CC cells and no binding between EMR1 and JAK2 in EMR1^KO^ CC cells. Upon coculture with macrophages, EMR1 IP binding with the JAK2 protein increased in EMR1^+^ CC cells compared to culture without macrophages. In contrast, EMR1 expression and EMR1 Co-IP binding with JAK2 were observed in EMR1^KO^ CC cells. These findings suggest that macrophages can induce EMR1 expression and facilitate its binding with JAK2 in CC cells (Figure 4L). Overall, our results provide compelling evidence that the upregulation of EMR1 in cancer cells by macrophages influences critical cellular functions, with mechanistic involvement of JAK2/STAT1,3 signaling in CC.

#### 2.2.3. Effect of JAK2/STAT3 Pathway Inhibitor (SD1008) on the Molecular and Biologic Characteristics of CC Cells According to EMR1 Expression

**Figure 5:**
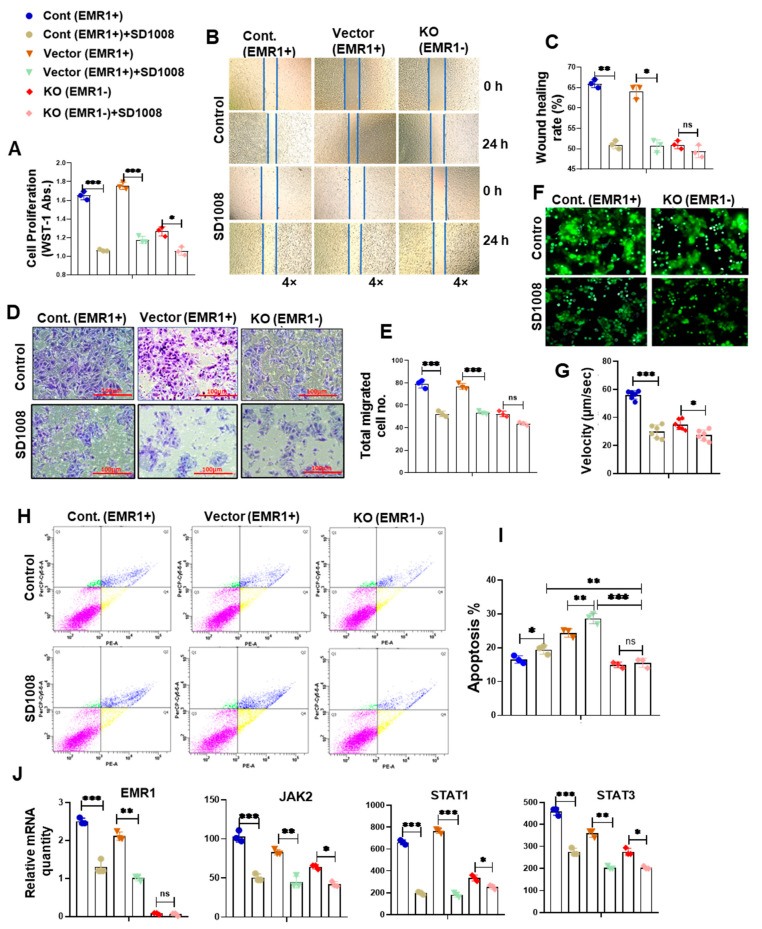
**Effects of a JAK2/STAT3 pathway inhibitor on the EMR1-related molecular changes and biological behavior of CC cells in vitro.** (**A**–**G**) The WST-1 (**A**), scratch wound healing (**B**,**C**), and Transwell migration assays (**D**,**E**) and live cell imaging using NanoLive assay (**F**,**G**) revealed reduced proliferation, migration, and mobility of EMR1^+^CC cells; however, no significant changes were found in EMR1^KO^ CC cells after treatment with a JAK2/STAT3 inhibitor (SD1008). (**H**,**I**) SD1008-induced apoptosis of EMR1^+^CC cells was detected via Annexin-V/7-AAD staining and flow cytometry; however, no significant changes were observed in EMR1^KO^ CC cells after SD1008 treatment. (**J**) qRT-PCR was performed to determine the relative mRNA expression between EMR1^+^ and EMR1^KO^ CC cells after SD1008 treatment. The results represent two independent experiments, each comprising three replicates, with statistical analysis conducted based on these three replicates. Error bars represent SD. Significance levels: * *p* < 0.033; ** *p* < 0.002; and *** *p* < 0.001, determined via Bonferroni-corrected one-way ANOVA relative to the control group. Abbreviations: CC-cont: colon cancer cell-control, KO: knockout, Abs: absorbance, SD: standard deviation, ns: not significant; PE-A: PE-Annexin-V; and PerCP-Cy5-5-A: 7-aminoactinomycin D (7-AAD).

**Figure 6:**
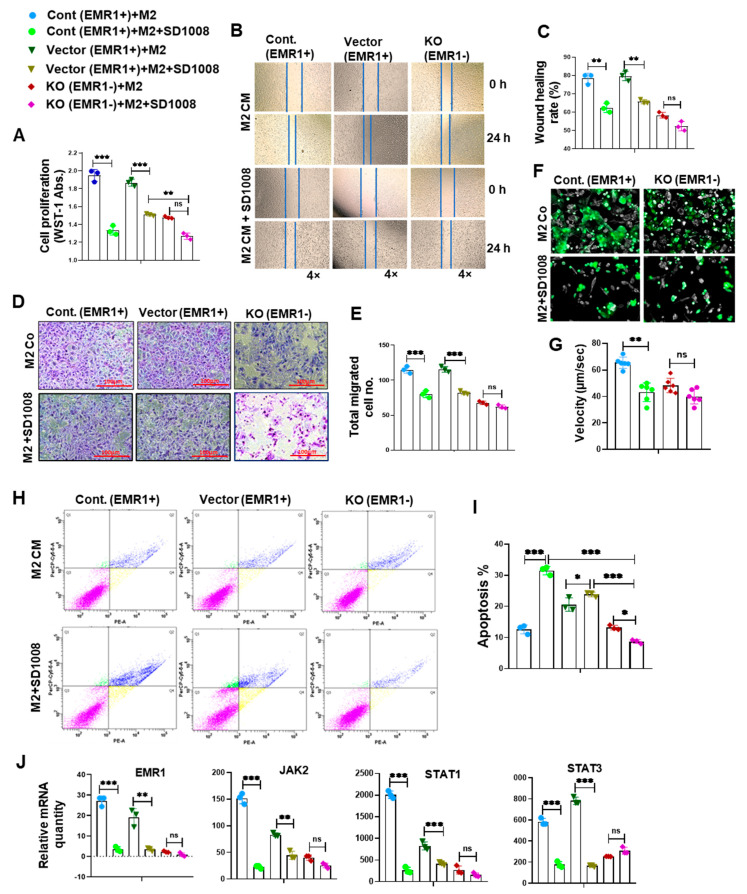
**Effects of a JAK2/STAT3 pathway inhibitor on the molecular changes and biological behavior of CC cells cocultured with macrophages in vitro.** (**A**–**G**) The WST-1 (**A**), scratch wound healing (**B**,**C**), and Transwell migration assays (**D**,**E**) and live cell imaging using NanoLive assay (**F**,**G**) revealed reduced proliferation, migration, and mobility of EMR1^+^ CC cells; however, no significant changes were observed in EMR1^KO^ CC cells after treatment with a JAK2/STAT3 inhibitor (SD1008) and coculture with macrophages. (**H**,**I**) Combined (SD1008 and M2 CM) treatment-induced apoptosis of EMR1^+^ CC cells compared to EMR1^KO^ CC cells was analyzed via Annexin-V/7-AAD staining and flow cytometry. (**J**) qRT-PCR was performed to determine the relative mRNA expression between EMR1^+^ and EMR1^KO^ CC cells after SD1008 treatment and coculture with macrophages. The results represent two independent experiments, each comprising three replicates, with statistical analysis conducted based on these three replicates. Error bars represent SD. Significance levels: * *p* < 0.033; ** *p* < 0.002; *** *p* < 0.001, determined via Bonferroni-corrected one-way ANOVA relative to the control group. Abbreviations: SD: standard deviation, ns: not significant; PE-A: PE-Annexin-V; and PerCP-Cy5-5-A: 7-aminoactinomycin D (7-AAD).

To determine JAK2/STAT3 inhibitor (SD1008) effect on cell viability, the cells were treated with different concentrations of the SD1008 inhibitor for 48 h. In related experiments, 10 µg/mL of SD1008 was administered for direct cancer cell treatment and in the coculture systems. At this concentration, the viability of macrophage and CC cells were greater than 70% (Supplementary Figure S4A,B). Treatment with SD1008 led to a more significant reduction in EMR1, JAK2, STAT1 and STAT3 mRNA expression in EMR1^+^ CC cells compared to EMR1^KO^ cells (Figure 5J and Figure 6J). Functional assays, including the WST-1, scratch wound healing, Transwell migration, and live cell mobility assays, revealed a significant decrease in the proliferation, migration, and mobility of EMR1^+^ CC cells following SD1008 treatment and coculture with or without macrophages. No significant changes were observed in EMR1^KO^ cells (Figure 5A–G and Figure 6A–G). Treatment with SD1008 also induced a more significant increase in the apoptosis of EMR1^+^ CC cells; however, no apparent effect was found in EMR1^KO^ cells (Figure 5H,I). After combined treatment with M2 CM and SD1008, the apoptotic rate (%) of EMR1^+^ cells were significantly higher than that of EMR1^KO^ cells. (Figure 6H,I). These findings indicate the specific and pronounced effects of EMR1 on JAK2/STAT3 pathway inhibition, affecting key functional aspects, such as proliferation, migration, mobility, and apoptosis. These differential responses highlighted the potential of targeted therapies for EMR1 in CC, especially in the presence of macrophages.

## 3. Discussion

In this study, we performed spatial transcriptomic profiling using GeoMx-DSP on surgically resected FFPE tissues of pT3 MSI-H CC to assess the molecular signaling mechanism according to LNM and EMR1 expression. In addition, an in vitro assay was conducted using a coculture system to determine the effect of macrophages on CC cells owing to EMR1 and the associated signaling pathway. Given the heterogeneity in EMR1 expression revealed by IHC staining, spatial profiling was selected to reveal the intricate spatial organization and specific gene expression patterns within tissues. Spatial transcriptomics analysis enables deeper comprehension of the cellular milieu underlying disease pathophysiology [19]. As a pivotal pioneer in this field, the Nanostring GeoMx DSP platform facilitates comprehensive exploration of the whole-genome spatial transcriptome, encompassing over 18,000 human genes, in a high-throughput manner, from FFPE and frozen specimens [20,21]. The Nanostring GeoMx transcriptomic data must be meticulously processed and analyzed to discern DEGs with heightened reliability, thereby enhancing our insights into the spatial transcriptome profiles of target tissues.

Previously, we found that EMR1 is a risk factor for LNM in CC [6]. In the current analysis of transcriptomic alterations based on LNM, CC with LNM was found to exhibit distinct changes in gene expression profile, including upregulated EMR1 compared to CC without LNM, consistent with the results of other LNM-based transcriptomic studies [22,23]. These investigators described biological distinctions between LNM and tumor deposits, such as upregulation of matrix remodeling, cell adhesion/motility, and epithelial–mesenchymal transition, and its association with poor prognosis in CRC [22]. Another study on spatial profiling of CC metastasis revealed that the TME characterized by spatially dependent immune cell-specific expression patterns can predict metastasis, including LNM [23]. The coordinated immune response was also elucidated and key biomarkers, such as GZMB, PD-L1, CD8, CD3, CD56, FOXP3, CD66b, and fibronectin, were identified [23]. Our results confirmed a significant increase in ADGRE1 (EMR1) expression in the LNM group, supporting and reinforcing our earlier observations.

We explored transcriptomic alterations based on EMR1 expression that can influence LNM in CC. Numerous biological pathways, including the JAK/STAT signaling pathway with inflammatory response, cytokine–chemokine mediated signaling, and antigen processing and presentation, and their relevant DEGs (such as TNFSF10, IL-32, IL22RA1, CCL-5, CXCL-1, 2, 3, 11, and 16, HLA-DMA, HLA-DPA/B, HLA-DRA/B, and HLA-F) were enriched in CC with EMR1^H/L^ and LNM^+^ compared to EMR1^N^ and LNM^−^ CC (Figure 2D,H–M). Based on substantial evidence that has been recently obtained, hyperactivated JAK/STAT signaling contributes to tumorigenesis, tumor growth, cancer cell viability, and metastasis in diverse solid tumors, including CC [24,25]. Clinical associations have been established between JAK/STAT signaling and CRC parameters, such as clinical stage, tumor infiltration depth, LNM, and poor prognosis [26]. In addition, the JAK/STAT signal transduction pathway plays regulatory roles in cytokine receptor signaling, chemokine-mediated signaling, inflammatory response, and antigen processing pathways, which can influence the progression of different types of cancer, including CC [27,28]. According to Zhang et al., the TNF-α/JAK/STAT signaling pathway contributes to oncogenesis by promoting the cell cycle, proangiogenic activity, and neovascularization of tumors, and preventing the apoptosis of human CRC cells [29]. Mesenchymal stem cells derived from human CRCs facilitate colorectal cancer progression via the IL-6/JAK2/STAT3 signaling pathway [30]. YH Liang et al. identified a robust association between the IFN-γ/JAK/STAT1 signaling pathway and principal immunogenic response elicited through the activation of MHC class I in human CC cells [31]. Moreover, studies indicate that JAK/STAT signaling pathway-related factors improve MHC class II immunoreactivity [32,33], which can promote LNM, particularly in breast cancer [34]. However, the relationship between EMR1 and JAK/STAT signaling and cytokine (TNFSF10, IL-32, IL-22RA1), chemokine (CCL-5, CXCL-1, 2, 3, 11, and 16), and MHC-I/II (HLA-DMA, HLA-DPA/B, HLA-DRA/B, HLA-F)-mediated signaling in CC progression has not been previously reported. Based on our findings, when EMR1 binds to its ligand, JAK/STAT signaling is activated, potentially inducing the production of cytokines and chemokines, enhancing MHC class I/II immunoreactivity, and consequently fostering LNM in the progression of CC.

Previously, we reported that EMR1 upregulation in cancer cells was induced by macrophages in an in vitro coculture system [6]. Considering the effect of macrophages on EMR1 expression in CC cells, an nCounter RNA sequencing pan-cancer immune panel was used to assess the role of EMR1 in CC cells with macrophages and the associated signaling pathway. The nCounter RNA sequencing pan-cancer immune panel was designed to provide insights into the immune activities of different cancer types, enabling an analysis of the expression of a curated set of genes related to immune response and function across different cancers. Moreover, a deeper understanding of the complex interplay between the immune system and cancer can be gained. In the current study, gene expression analysis using the nCounter system revealed that JAK/STAT signaling, cytokine–chemokine, and antigen presentation signaling were enriched in EMR1^+^ CC cells compared with EMR1^KO^ CC after coculture with macrophages, thereby aligning with the GeoMx profiling results. In addition, qRT-PCR and Western blotting confirmed that macrophage-induced upregulation of EMR1 expression in CC cells coincided with elevated expression of JAK2/STAT1,3. As their direct combination has not been reported in the literature, we performed Co-IP experiments to verify the interaction of two proteins. In this study, we observed that macrophages can induce EMR1 expression in EMR1^KO^ cells also, and inhibition of the JAK2/STAT3 pathway can suppress EMR1 expression in cancer cells, suggesting a potential link between EMR1 activation and the transcriptional regulation mediated by JAK2/STAT3 signaling in cancer cells. Further investigations are warranted to confirm this hypothesis. By investigating the biological changes, we found increased proliferation, migration, motility and decreased apoptosis of EMR1^+^ CC cells after coculture with macrophages compared to the control. Conversely, inhibition of JAK2/STAT3 signaling suppressed this phenomenon along with EMR1 expression in EMR1^+^ CC cells cocultured with or without macrophages. However, EMR1^KO^ CC cells exhibited negligible alterations in proliferation, migration, motility and apoptosis in the presence and absence of macrophages, compared to control cells. Treatment with the JAK2/STAT3 (SD1008) inhibitor led to minimal changes in the expression of JAK2/STAT1,3 in EMR1^KO^ CC cells compared with the control. Recently, numerous studies have indicated that overly activated JAK/STAT signaling promotes the proliferation, migration, and invasion of cancer cells in CC, whereas the inhibition of JAK2/STAT3 signaling suppresses cancer progression [35,36,37,38,39]. According to Wang et al., circSPARC, a circular RNA, potentiates CRC cell migration and proliferation by modulating the JAK/STAT signaling pathway [35]. SphK1 has been reported as a promotor of cancer progression by activating the JAK/STAT pathway and enhancing S1PR1 expression in CC cells [36]. Yang et al. revealed the enhanced cellular proliferation and migration of CC cells, which were facilitated by FKBP14 via modulation of the IL-6/STAT3 signaling pathway [37]. Inhibition of JAK2/STAT3 signaling induces CRC cell apoptosis and cell cycle arrest, and suppresses tumor growth and invasion in CRC [38,39]. However, according to our findings, overexpression of EMR1 is not only associated with macrophages in the TME, but also the activation of JAK2/STAT1,3 signaling in cancer cells. To the best of our knowledge, this study is the first to explore the biological role of EMR1 in CC progression via activation of the JAK2/STAT1,3 signaling pathway. The observed interplay between EMR1, JAK2/STAT1,3 signaling, and macrophages suggests the potential role of EMR1 in CC progression and therapeutic response to the JAK2/STAT3 signaling inhibitor, especially in macrophage-rich tumors. Furthermore, considering the pivotal role of macrophages in EMR1-mediated cancer progression via the JAK2/STAT1,3 signaling pathway, future experiments employing primary monocytes/macrophages could provide valuable translational insights.

## 4. Materials and Methods

### 4.1. Ethics Approval

This study was approved by the Institutional Ethics Committee of Yonsei University, Wonju College of Medicine (CR-321336) and adhered to the principles outlined in the Declaration of Helsinki.

### 4.2. Whole Transcriptome Analysis Using GeoMx Digital Spatial Profiling (DSP)

#### 4.2.1. Tissue Sample Preparation and Procedures for GeoMx DSP

Spatial RNA profiling was performed using NanoString’s GeoMx DSP platform (Seattle, WA, USA) with experimental management performed by Ebiogen (Seoul, Republic of Korea). Formalin-fixed paraffin-embedded (FFPE) tissues from 14 patients with surgically resected pT3 microsatellite unstable (MSI-H) CC (4 LNM+ and 10 LNM-), registered at the Wonju Severance Christian Hospital BioBank (Wonju, Republic of Korea) were collected between 2018 and 2020. Immunohistochemical (IHC) detection of EMR1 expression in paraffin-embedded tissue sections was performed using an automated staining platform (BenchMark XT; Ventana Medical Systems, Tucson, AZ, USA), according to the established protocols [40]. The specimen slides were incubated with primary antibodies targeting EMR1 (Cell Signaling Technology, DA, MA, USA) in an autostainer for 2 h at 37 °C, according to the UltraView Universal DAB Detection Kit (BenchMark XT, AZ, USA). The specimens were subsequently analyzed using a BX51 microscope (Olympus, Tokyo, Japan).

Based on EMR1 expression, one or two tissue cores (size, 3 mm) from the center of the tumor were used to construct a tissue microarray (TMA) block. Subsequently, 4 μm FFPE tissue sections from the TMA block were stained with PanCK and Syto13 DNA for immunofluorescence analysis. The regions of interest (ROIs) were selected according to EMR1 expression, with 66 ROIs chosen based on EMR1 expression intensity (high, low, and negative). In terms of homogenous EMR1 expression, three ROIs were selected; however, for heterogenous EMR1 expression, 3 ROIs were selected from two distinct expression area, totaling 6 ROIs. The tissue segmentation overlay was used to visualize PanCK+ segments in a selected 500 µm width. The slides were hybridized with probes targeting 18,675 genes and loaded onto GeoMx DSP after morphology marker staining. UV exposure induced the release of indexing oligos that were collected using a microcapillary and deposited into a 96-well plate. Sequencing libraries were generated via PCR, pooled, purified, and sequenced using an Illumina NovaSeq6000 for RNA expression quantification.

#### 4.2.2. Dimension-Reduction Using t-Distributed Stochastic Neighbor Embedding (t-SNE)

Dimensionality reduction in the expression matrix was performed by applying t-SNE using the R package by NanoString. Differentially expressed genes (DEGs) in the EMR1 high or low group compared to the negative group and lymph node positive and negative groups were subjected to filters based on Benjamini–Hochberg adjusted *p*-value < 0.05 and an absolute log2 fold change > 1.

#### 4.2.3. Functional Enrichment Analysis

Gene Ontology (GO) and Kyoto Encyclopedia of Genes and Genomes (KEGG) enrichment analyses were performed using the NanoString R package. Significantly enriched terms were determined based on a statistical threshold of *p* < 0.05. Gene set enrichment analysis (GSEA) was used to identify gene sets markedly associated with specific pathways in the sequencing data. This analysis was conducted using GSEA 4.2.3 software, employing the “HALLMARK” gene set collections (comprising 50 sets) derived from the Molecular Signatures Database (version 7.1). The normalized enrichment score (NES), which accounts for the size of each gene set, was computed, and a false discovery rate <0.05 was considered to indicate statistically significant enrichment.

### 4.3. In Vitro Validation of EMR1-Related Differential Gene Expression in CC Cells Using a Coculture System with Macrophages

#### 4.3.1. Cell Culture and Reagents

THP-1 human monocyte and HCT15 CC cell lines were procured from the Korean Cell Line Bank. The cells were cultured in Roswell Park Memorial Institute (RPMI)-1640 medium (HyClone, Logan, UT, USA) supplemented with 10% fetal bovine serum (FBS), 1% penicillin (100 U/mL), and streptomycin (100 mg/mL; Gibco, Grand Island, NY, USA) at 37 °C in a humidified atmosphere with 5% CO2.

#### 4.3.2. Generation of EMR1KO HCT15 CC Cells with the CRISPR-Cas9 Gene Editing System, and Validation via Western Blotting and Flow Cytometry

Guide RNA sequences for CRISPR-Cas9 were designed using CRISPR design website, accessed on 19 October 2021 (http://crispr.mit.edu/). The insert oligonucleotides for human EMR1 gRNA were as follows:

EMR1sgRNA.1F: 5′-CACCGTCCACTGTATTGGTGCAGG-3′

EMR1sgRNA.1R: 5′-AAACCCTGCACCAATACAGTGGAC-3′

EMR1sgRNA.2F: 5′-CACCGCAGGTGCAAAAGTAGCTCCC-3′

EMR1sgRNA.2R: 5′-AAACGGGAGCTACTTTTGCACCTGC-3′

Guide RNAs were annealed and integrated into the Lenticrispr V2 vector, with the GFP lentivirus as a reference. Lenticrispr V2 was transfected into HEK-293T cells and a medium change was performed at 24 h. The lentivirus obtained after three days was concentrated via centrifugation. HCT15 cells were transfected with CRISPR-Cas9 or GFP lentiviruses. The transfection efficiency was validated by counting the number of GFP-positive cells. EMR1KO cells were derived from single-cell colony culture. The cells were trypsinized, centrifuged, and resuspended in FBS Stain Buffer (BD Pharmingen™, San Diego, CA, USA). EMR1-PE (Santa Cruz Biotechnology, Santa Cruz, CA, USA), a monoclonal antibody, was introduced and incubated in the dark for 60 min at 4 °C. Following washing, cells were resuspended in FBS Stain Buffer for flow cytometry using a BD FACS Aria III (BD Bioscience, San Jose, CA, USA).

#### 4.3.3. Polarization of Macrophages

THP-1 monocytes were cultured and treated with 200 nM phorbol-12-myristate-13-acetate for 48 h to induce M0 macrophage differentiation. Macrophage polarization was induced by the addition of M2-polarizing reagents (20 ng/mL interleukin (IL)-4 + 20 ng/mL IL-13; Invitrogen, WA, MA, USA) and incubation at 37 °C for 48 h. Following trypsinization, the cells were centrifuged, resuspended in FBS Stain Buffer, and labeled with CD86-APC, CD163-PE, and CD206-FITC (Abcam, Cambridge, UK) monoclonal antibodies. After a 60 min incubation in the dark at 4 °C, the cells were washed, resuspended in FBS Stain Buffer, and subjected to flow cytometry using a BD FACS Aria III.

#### 4.3.4. Coculture Procedures

Transwell^®^ plates with 0.4 μm pores (Corning, NY, USA) were used for coculture. Macrophages were initially seeded in the upper chamber while EMR1+ or EMR1KO CC cells (1 × 105 cells/mL) were seeded in the lower chamber for 48 h. Following incubation, mRNA and protein analyses were conducted using lysed cancer cells collected from the lower chamber.

#### 4.3.5. Exploring EMR1-Related RNA Expression in CC Cells after Coculture with Macrophages Using an nCounter System

Gene expression analysis was performed using the nCounter gene expression platform (NanoString, Seattle, WA, USA) with the NanoString Pan-Cancer IO 360 panel, which covers 750 genes relevant to tumors, TME, and immune response pathways. For each sample, 100 ng of total RNA was hybridized overnight with three biotinylated capture probes and five reporter probes containing fluorescent barcodes. The hybridized samples underwent high-sensitivity processing at the NanoString nCounter preparation station and were scanned at high resolution using an nCounter Digital Analyzer.

PCR (Kyratec Super Cycler) was initiated, and the mRNA hybridization file was loaded at 65 °C. The hybridization conditions were verified, and the run was initiated when the Top Heater reached 72 °C or 12 strip tubes were inserted. The RNA concentration of the sample was measured using a Denovix ds 11 spectrophotometer, and the input volume was calculated. The samples (sample + DEPC water) were dispensed into 12-strip tubes. The Reporter CodeSet and Capture ProbeSet reagents were thawed, mixed, and dispensed into tubes. The master mix was created using a hybridization buffer and dispensed into the tubes. The reaction mixture was incubated for at least 18 h at 65 °C.

#### 4.3.6. Exploring EMR1-Related Protein Expression after Coculture with Macrophages Using Western Blotting

Protein extraction and Western blotting were performed as described in our previous publications [41,42]. In this study, the primary antibodies targeted EMR1 (F4/80) and β-actin (Santa Cruz Biotechnology, Santa Cruz, CA, USA), and JAK2, STAT1, p-STAT1, STAT3, and p-STAT3 (Cell Signaling Technology, Danvers, MA, USA). After incubation with the primary antibody, cells were incubated with peroxidase-conjugated secondary antibodies (1:2000; Santa Cruz Biotechnology, Santa Cruz, CA, USA; Cell Signaling Technology, Danvers, MA, USA).

#### 4.3.7. Exploring EMR1-Related mRNA Expression after Coculture with Macrophages Using Real-Time Quantitative Reverse Transcription Polymerase Chain Reaction (qRT-PCR)

Total RNA was extracted from the coculture system using TRIzol^®^ Reagent, according to the manufacturer’s instructions. cDNA was synthesized from 1 µg of total RNA using the QuantiTect^®^ Reverse Transcription Kit. qRT-PCR was performed in 384-well plates using the Fast SYBR Green Master Mix. GAPDH served as the internal control, and the QuantStudio™ 6 Flex System was used for real-time PCR with a 40-cycle protocol. Gene expression was quantified using the Ct method. The following primer sequences were used in this experiment:

EMR1. RT Forward-5′-GCATGACACTGGCATCTTTTTG-3′

EMR1. RT Reverse-5′-AATGTCTAAGTATTCCGTCCGAACA-3′

JAK2. RT Forward-5′-CCAGATGGAAACTGTTCGCTCAG-3′

JAK2. RT Reverse-5′-GAGGTTGGTACATCAGAAACACC-3′

STAT1. RT Forward-5′-ATGGCAGTCTGGCGGCTGAATT-3′

STAT1. RT Reverse-5′-CCAAACCAGGCTGGCACAATTG-3′

STAT3. RT Forward-5′-GACCAGATGCGGAGAAGCAT-3′

STAT3. RT Reverse-5′-CTCTTCCAGTCAGCCAGCTC-3′

GAPDH. Forward: 5′-AGGTCGGTGTGAACGGATTTG-3′

GAPDH. Reverse: 5′-TGTAGACCATGTAGTTGAGGTCA-3′

#### 4.3.8. Exploring EMR1-Related Protein-Protein Interaction after Coculture with Macrophages Using Co-Immunoprecipitation (Co-IP)

A total of 0.5 mg of protein was used for each pull down reaction with EMR1 (F4/80) (Santa Cruz Biotechnology, Santa Cruz, CA, USA) and JAK2 (Cell Signaling Technology, Danvers, MA, USA) antibodies fused to protein A agarose (Cell Signaling Technology, Danvers, MA, USA) at 4 °C for 3 h. After washing with 1× PBS, the proteins were separated via sodium dodecyl sulfate–polyacrylamide gel electrophoresis (SDS-PAGE) and immunoblotted.

### 4.4. Evaluation of the Biological Changes of Cancer Cells Due to EMR1 Expression after Coculture with Macrophages

#### 4.4.1. Cell Proliferation Assay

CC cell proliferation was assessed using the WST-1 assay. Cells (5 × 103 cells/well) were seeded in 96-well plates in RPMI supplemented with 10% FBS and incubated at 37 °C with 5% CO2. After treatment with M2 macrophage-conditioned medium (M2 CM) and 10 µM SD1008 (a JAK2/STAT3 inhibitor, Tocris Bioscience, Minneapolis, MN, USA) for 48 h, 10 mL of the WST-1 reagent (Roche, Indianapolis, IN, USA) was added and the mixture was further incubated for 1 h. Absorbance was measured at 450 nm using an ELISA reader.

#### 4.4.2. Transwell Migration Assay

Cells (1 × 106 cells in 700 µL RPMI with 1% FBS) were placed in the upper chamber of a Corning Transwell with 8 μm pores. After 48 h of coculture with M2 macrophages and SD1008 treatment, migrated cells on the reverse side were fixed, stained with HE, and counted under an inverted microscope.

#### 4.4.3. Wound Healing Assay

Mitomycin C (0.1 mg/mL, Sigma-Aldrich)-treated cells were scratched using a pipette tip (200 µL yellow tip) and then cells were cultured with serum free media to prevent the proliferation of cells. After 24 h of treatment with or without M2 CM and SD1008 (a JAK2/STAT3 inhibitor), cell migration images were acquired using the EVOS^®^ XL Core Imaging System (Invitrogen, Thermo Fisher Scientific) at 4× magnification and assessed using ImageJ software. The wound healing rate (%) was calculated as follows: [(area at 0 h—area at 24 h)/(area at 0 h)] × 100.

#### 4.4.4. Cell Motility Assay

NanoLive 3D Cell Explorer was used to analyze the movement of live cells. Cells were seeded at a density of 1 × 104 cells/well in a glass-bottom dish and incubated overnight at 37 °C with 5% CO2. Cancer cells, identified using CellTracker Green CMFDA, were cocultured with macrophages with or without SD1008 treatment (JAK2/STAT pathway inhibitor) for 24 h. To assess cancer cell movement, live cell images were captured every 6 min per cycle and a total of 80 cycles were performed over 8 h using the FITC fluorescence field of the NanoLive 3D Cell Explorer and cell movement was assessed using ImageJ software (Fiji ImageJ-Win 64).

#### 4.4.5. Apoptosis Assay

The apoptosis assay was performed as described in our previous report [41]. Briefly, the PE-Annexin-V apoptosis detection kit I (BD Biosciences, San Diego, CA, USA) was used, according to the manufacturer’s instructions. The cells were harvested, washed twice with cold PBS, and resuspended in a binding buffer. Thereafter, the cells were stained with PE-Annexin-V and 7-aminoactinomycin D (7-AAD) for 15 min at room temperature in the dark and then analyzed without washing using a flow cytometer (FACSAria III, BD Biosciences) within 1 h after the staining.

### 4.5. Statistical Analysis

Statistical analyses were performed using ExDEGA graphic software (Ebiogen, Seoul, Korea) and Prism (version 8.0, GraphPad Software, La Jolla, CA, USA). ExDEGA was used to process raw data and generate graphics for visualization. Genes with ≥1.3-fold change were considered statistically significant. One-way analysis of variance results for the in vitro coculture variables are presented as mean ± standard deviation. Statistical significance was indicated by *p* < 0.05 in all cases.

## 5. Conclusions

Our transcriptomic analysis provides valuable insights into the molecular mechanisms and biological implications of EMR1 expression in CC. The identified interplay of EMR1 with JAK/STAT signaling and macrophages enhances our understanding of the molecular mechanisms involving macrophages and intracellular EMR1 expression in CC, and their potential involvement in cancer progression and therapeutic response.

## Figures and Tables

**Table 1:** Patient demographic data.

Parameters			LNM+	LNM−
Age	≤50:>50		0:4	1:9
Sex	Male:Female		1:3	5:5
Location of CC	Right:Left		4:0	9:1
EMR1	High:Negative		1:0	2:3
	Heterogeneous	L/N	0	2
		H/L	2	3
		H/N	1	0

Abbreviations: LNM, lymph node metastasis; H = high, L = low, N = negative.

## Data Availability

Data are contained within the article and Supplementary Materials.

## Supplementary Materials

The supporting information can be downloaded at: https://www.mdpi.com/article/10.3390/ijms25084388/s1.
