# Supplementary material for: Upregulation of EMR1 (ADGRE1) by Tumor-Associated Macrophages Promotes Colon Cancer Progression by Activating the JAK2/STAT1,3 Signaling Pathway in Tumor Cells
Source: Int J Mol Sci. 2024 Apr 16;25(8):4388. doi: 10.3390/ijms25084388 (PMC11050366; doi:10.3390/ijms25084388)
Supplement: Supplementary file 1 [file ijms-25-04388-s001.zip › ijms-2900104-supplementary.pdf]

Supplementary materials

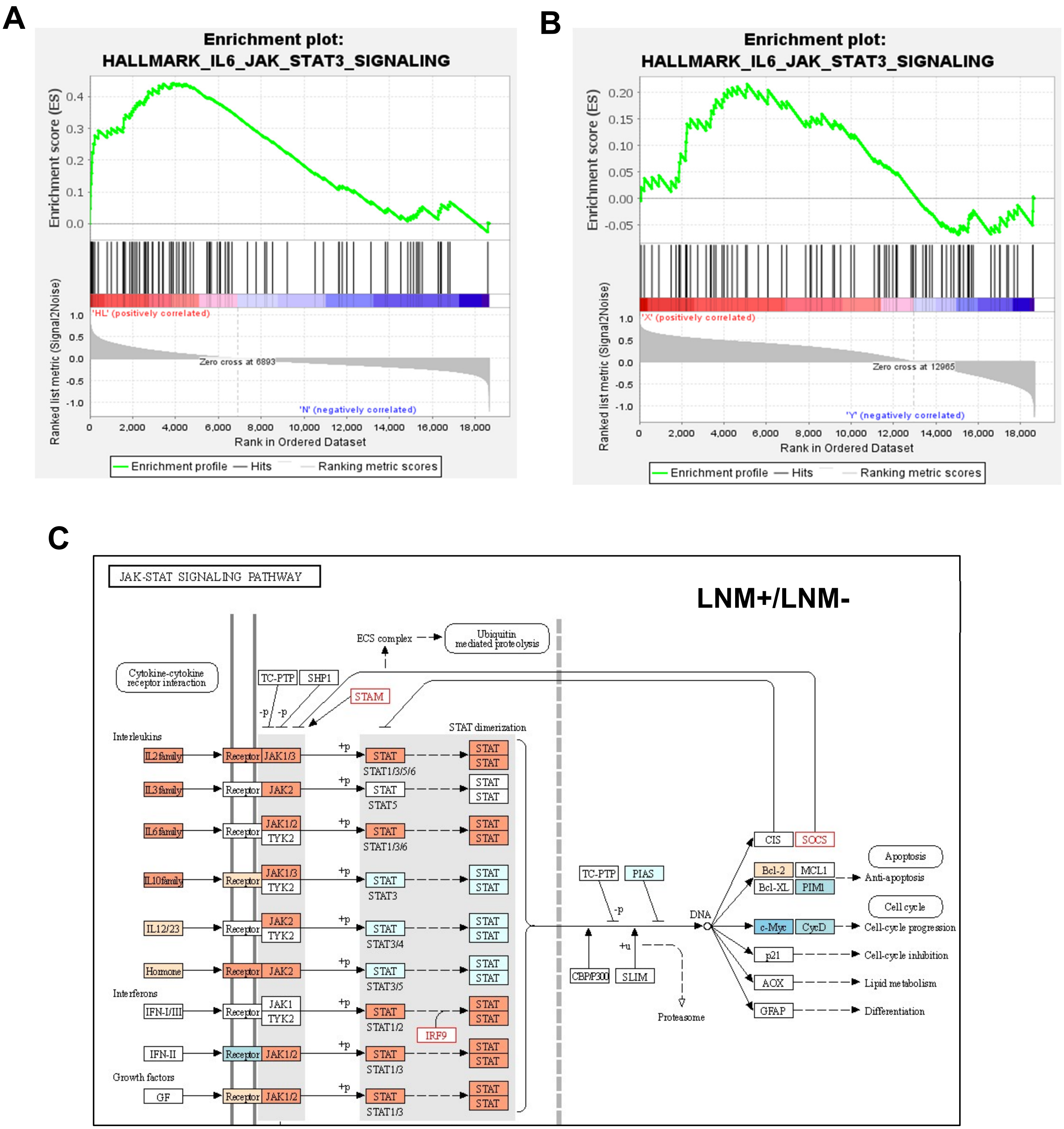

D

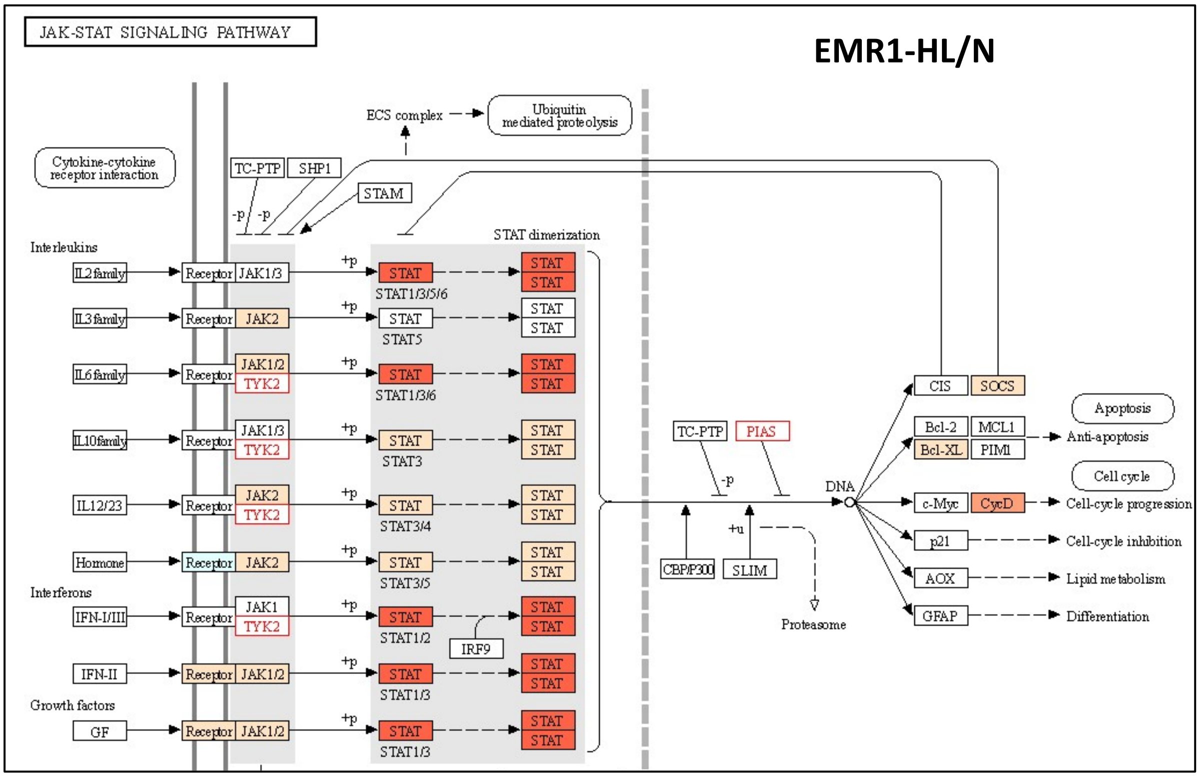

Supplementary Figure S1. Spatial transcriptomic analysis of CC according to EMR1 expression and LNM. A, B. GSEA enrichment plots showing JAK-STAT signaling pathway was enriched in the LNM+ vs LNM- group as well as EMR1-HL vs EMR1-N group. **C, D.** Map of the KEGG pathway "JAK-STAT SIGNALING PATHWAY" displaying genes with differentially expressed mRNA fragments in the LNM+ vs LNM- group as well as EMR1-HL vs EMR1-N group. The red color indicates upregulated and the blue color indicates downregulated mRNA. Here, X=LNM+, Y= LNM-. **Abbreviations:** LNM, lymph node metastasis; L=Low, H= High, N=Negative

A

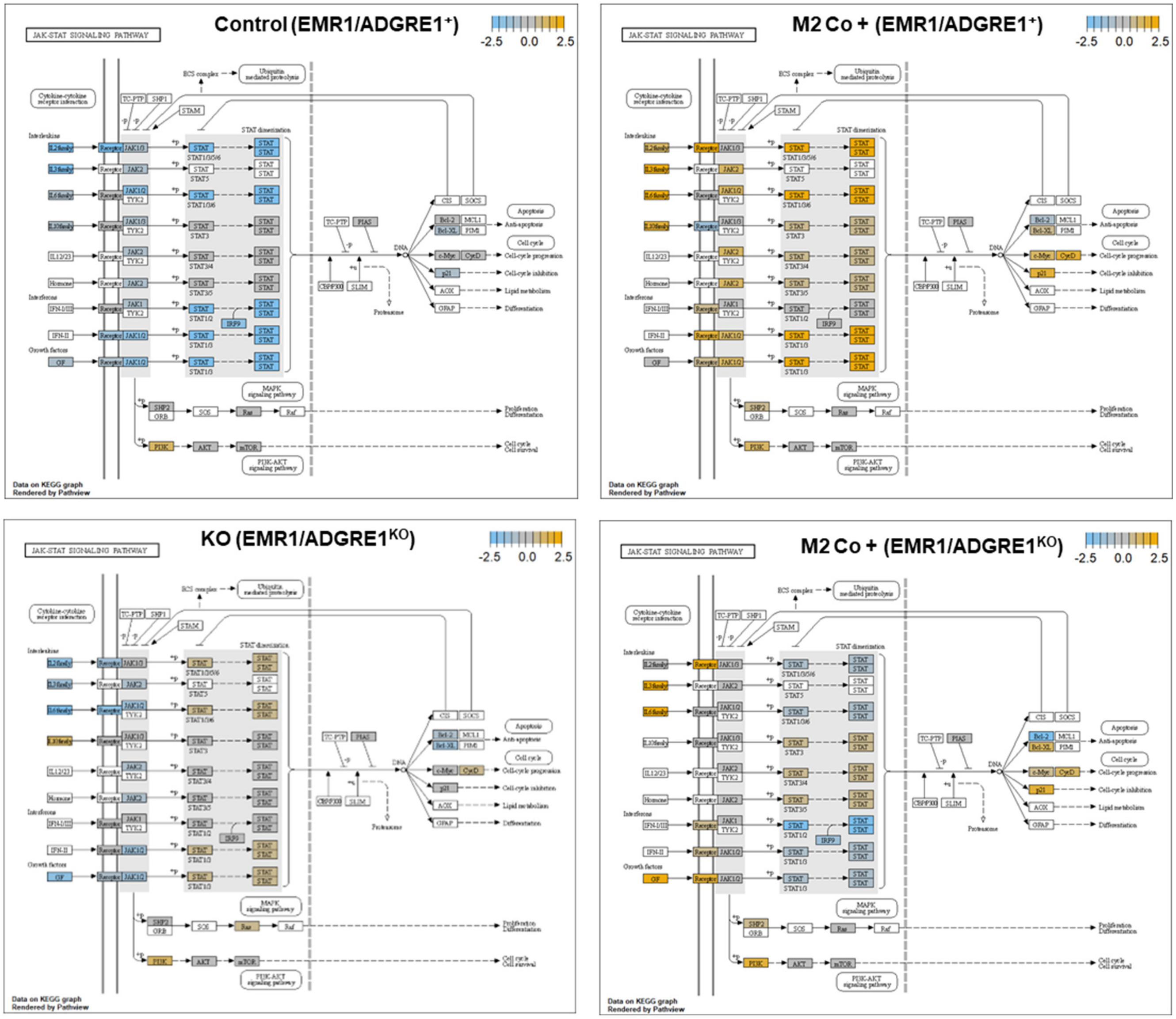

Supplementary Figure S2. Molecular changes between EMR1^{+} and EMR1^{KO} CC cells after coculture with macrophages in vitro. A. Map of the KEGG pathway "JAK-STAT SIGNALING PATHWAY" displaying genes with differentially expressed mRNA fragments. The yellow color indicates upregulated genes and the sky-blue color indicates downregulated genes.

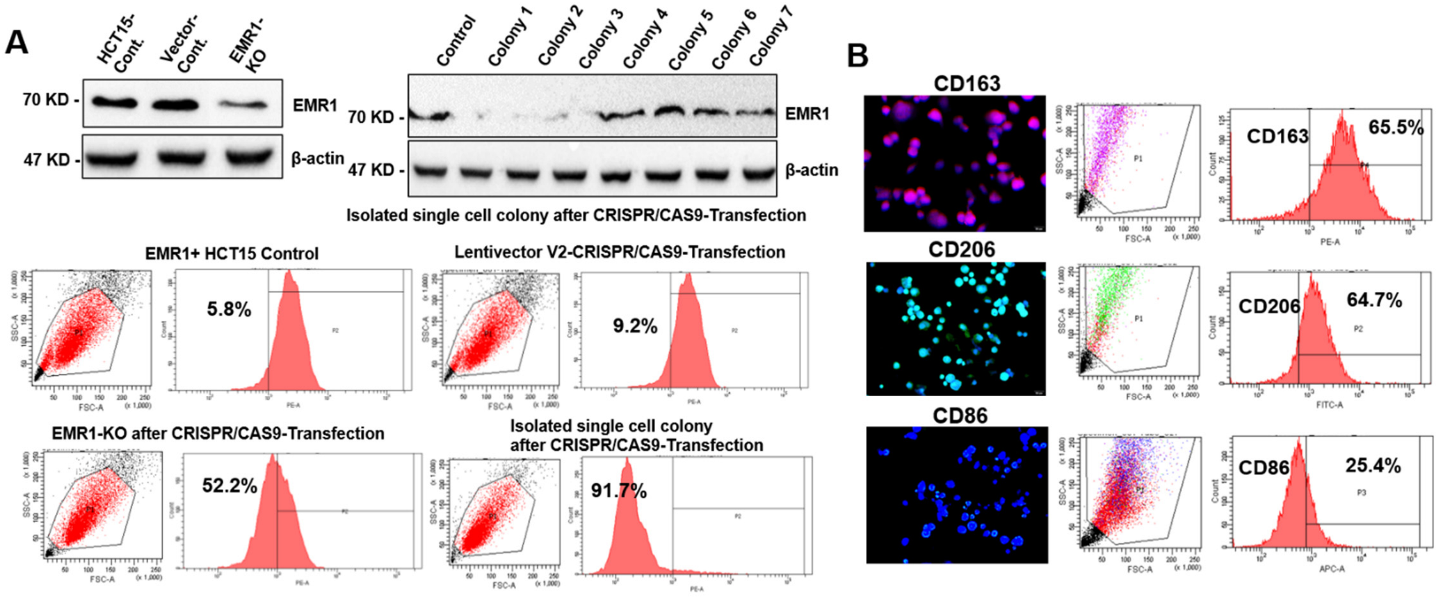

Supplementary Figure S3. EMR1 knockout and macrophage polarization validation results. A. Validation of CRISPR/Cas9-mediated EMR1 knockout by western blot and flow cytometry analysis**. B.** Validation of THP1-derived macrophage polarization by immunofluorescence and flow cytometry analysis. Abbreviations: CRISPR/Cas9.

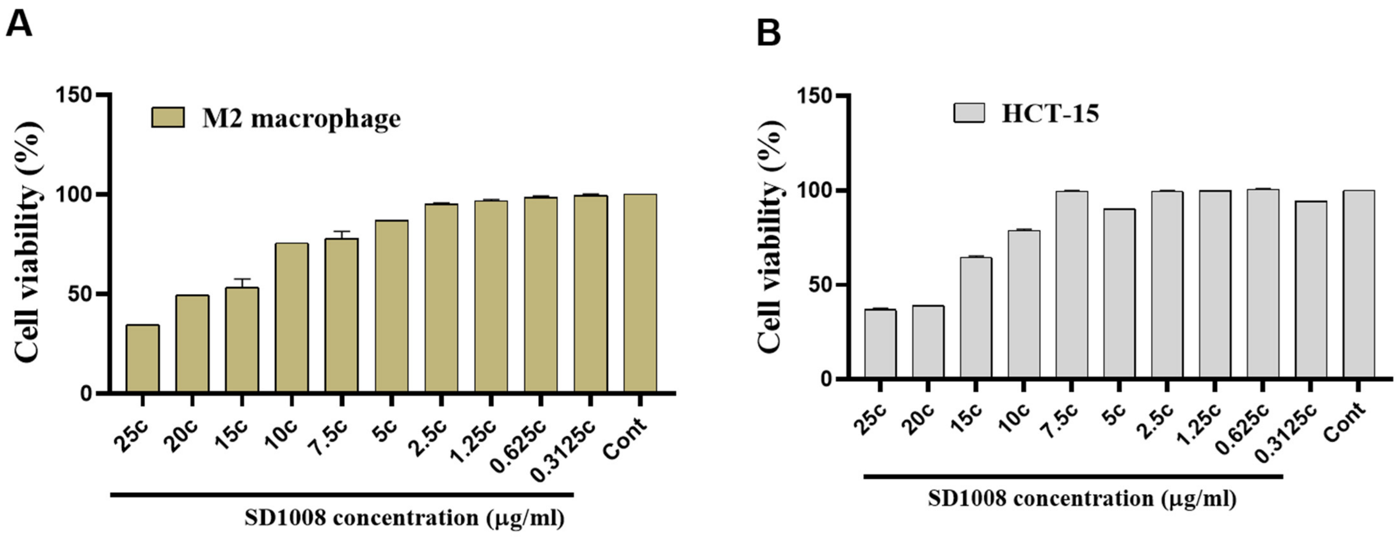

Supplementary Figure S4. Toxicity of JAK2/STAT3 inhibitor (SD1008) in macrophage and CC cells (HCT15). (**A-B**) The cells were treated with different concentrations of the SD1008 inhibitor for 48 h and toxicity was detected using a WST-1 reagent. Graphs represent data as means ± SD.
